# *In vitro* inhibition of *Pseudomonas aeruginosa* PAO1 biofilm formation by DZ2002 through regulation of extracellular DNA and alginate production

**DOI:** 10.3389/fcimb.2023.1333773

**Published:** 2024-01-10

**Authors:** Jiaze Dai, Wenying Luo, Fei Hu, Si Li

**Affiliations:** ^1^ Medical Laboratory Center, Affiliated Hospital of Guangdong Medical University, Zhan Jiang, Guang Dong, China; ^2^ General Medicine, Clinical Medicine, Kangda College of Nanjing Medical University, LianYun Gang, Jiang Su, China

**Keywords:** DZ2002, biofilm, extracellular DNA, alginate, *Pseudomonas aeruginosa*

## Abstract

**Introduction:**

*Pseudomonas aeruginosa* (*P. aeruginosa)* is a common pathogen associated with biofilm infections, which can lead to persistent infections. Therefore, there is an urgent need to develop new anti-biofilm drugs. DZ2002 is a reversible inhibitor that targets S-adenosylhomocysteine hydrolase and possesses anti-inflammatory and immune-regulatory activities. However, its anti-biofilm activity has not been reported yet.

**Methods and results:**

Therefore, we investigated the effect of DZ2002 on *P. aeruginosa* PAO1 biofilm formation by crystal violet staining (CV), real-time quantitative polymerase chain reaction (RT-qPCR) and confocal laser scanning microscopy (CLSM). The results indicated that although DZ2002 didn’t affect the growth of planktonic PAO1, it could significantly inhibit the formation of mature biofilms. During the inhibition of biofilm formation by DZ2002, there was a parallel decrease in the synthesis of alginate and the expression level of alginate genes, along with a weakening of swarming motility. However, these results were unrelated to the expression of *lasI, lasR, rhII, rhIR*. Additionally, we also found that after treatment with DZ2002, the biofilms and extracellular DNA content of PAO1 were significantly reduced. Molecular docking results further confirmed that DZ2002 had a strong binding affinity with the active site of S-adenosylhomocysteine hydrolase (SahH) of PAO1.

**Discussion:**

In summary, our results indicated that DZ2002 may interact with SahH in PAO1, inhibiting the formation of mature biofilms by downregulating alginate synthesis, extracellular DNA production and swarming motility. These findings demonstrate the potential value of DZ2002 in treating biofilm infections associated with *P. aeruginosa*.

## Introduction

*Pseudomonas aeruginosa* (*P. aeruginosa)* can often form biofilms in catheter-associated urinary tract infections (CAUTI) (Cole et al., 2014) and ventilator-associated pneumonia (VAP) (Maurice et al., 2018), especially in patients with cystic fibrosis (CF) (Ciofu et al., 2015), making the infections difficult to clear. Therefore, research and the development of new treatment strategies are of great significance in the prevention of *P. aeruginosa* infections caused by biofilms.

During the process of biofilm formation, bacteria can synthesize and secrete extracellular polymeric substances (EPS), such as proteins, extracellular polysaccharides (alginate, Psl, Pel) and extracellular DNA (eDNA) (Harimawan and Ting, 2016), which encase the microbial colonies and form complex biofilm structures. Thanks to the presence of biofilms, bacteria are given the ability to resist harsh environments, such as salt, acid and alkali, and ultraviolet light (Argyraki et al., 2017; Kovalchuk et al., 2021; Pezzoni et al., 2022). In general, bacteria with biofilms can exhibit higher resistance to antibiotics compared to planktonic bacteria (Silveira et al., 2021), making the infections difficult to clear. In order to combat biofilm-related infections, various new anti-biofilm technologies have been proposed, such as antimicrobial photodynamic therapy, phage therapy, and quorum sensing inhibitors (QSI) (Yin et al., 2022). However, most of these techniques require further validation through *in vivo* experiments and have certain limitations. Considering the prevalence of biofilm-associated microorganisms and the limited efficacy of current antibiotics, there is a need to explore non-toxic and effective anti-biofilm agents.

**Figure 1:**
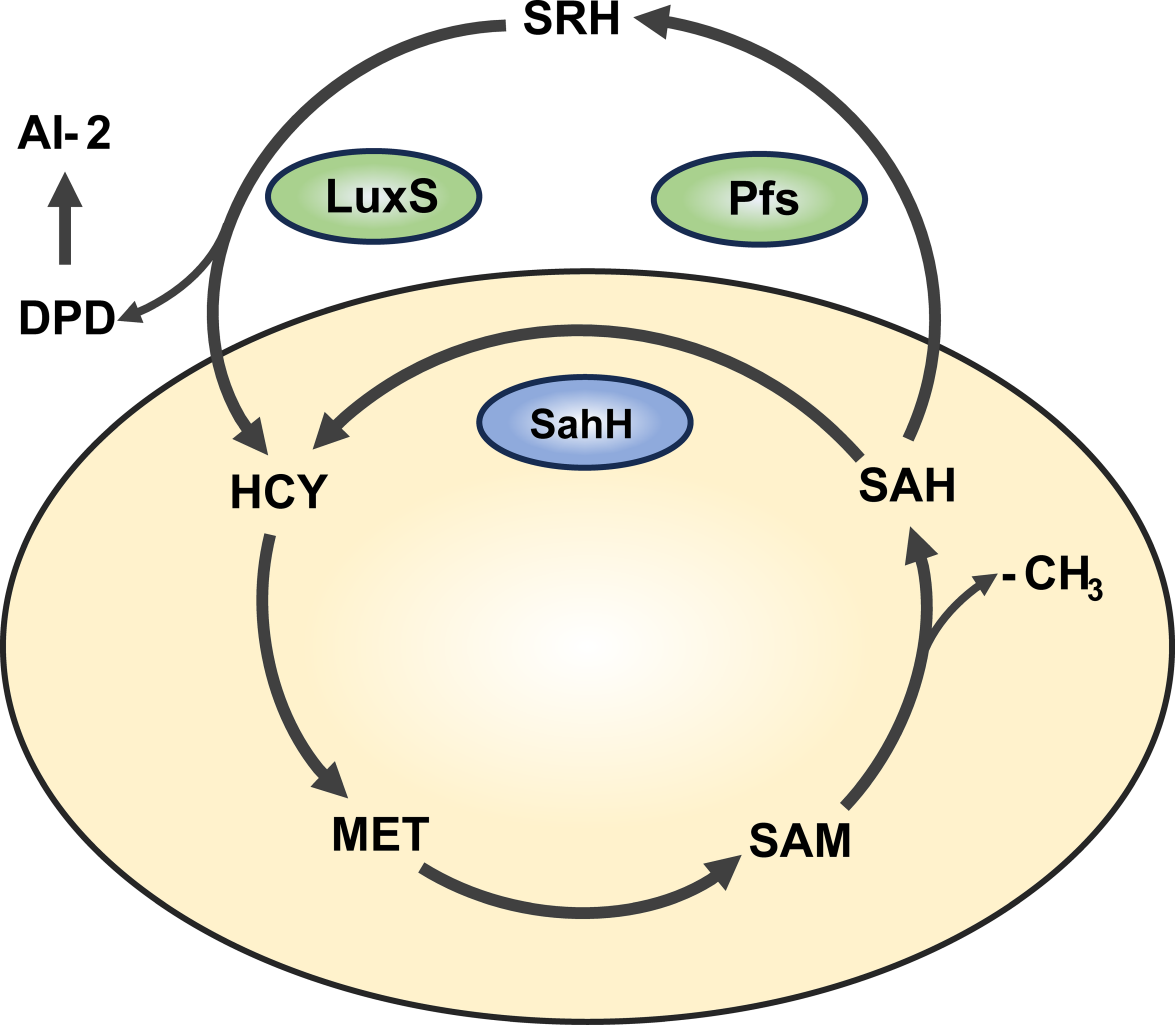
Activated methyl cycle pathway (AMC). The AMC pathway is derived based on previous reports (Parveen and Cornell, 2011). In this pathway, methionine (MET) is catalyzed by a series of enzymes to produce intermediates S-adenosine methionine (SAM), S-adenosine homocysteine (SAH), and activated methyl group (-CH3) for use by the body. In *P. aeruginosa*, SAH is hydrolyzed one-step to homocysteine (HCY) via the sahH pathway by S-adenosine homocysteine hydrolase (SahH), thus returning to the methyl cycle. This step is not generated by the Autoinducer-2 (AI-2). In bacteria such as *Escherichia coli*, SAH goes through the *LuxS/Pfs* two-step approach, first catalyzed by *LuxS* enzyme to produce intermediate products, and then catalyzed by Pfs enzyme to finally produce HCY, thus producing AI-2.

An increasing number of studies have shown that QS is an important mechanisms regulating biofilm formation (Kong et al., 2005; Liang et al., 2008). In *P. aeruginosa*, there are three major types of QS: *lasI/lasR, rhII/rhIR*, and *Pqs* (Flores-Percino et al., 2023). Additionally, the cross-species QS signal molecule, autoinducer-2 (AI-2), is known to mediate intercommunication between different bacterial species (Wang et al., 2023). Interestingly, in bacteria such as *Escherichia coli, Salmonella enterica*, and *Vibrio harveyi*, AI-2 is produced through a *LuxS/Pfs*-dependent activated methyl cycle (AMC) pathway (Taga et al., 2001; Sun et al., 2004). As an alternative pathway, *sahH*-dependent AMC exists in bacteria like *P. aeruginosa*, which does not produce AI-2 (Duan et al., 2003). The AMC process and AI-2 production are illustrated in **Figure 1**. Overexpression of *sahH* from *P. aeruginosa* in the *LuxS* mutant strain of *Streptococcus*, as observed elsewhere (Redanz et al., 2012; Hu et al., 2018), can restore biofilm-related phenotypes, indicating an association between methylation metabolism and biofilm formation. However, there is also study indicating that the methyl metabolism of avian pathogenic *Escherichia coli* has no effect on biofilm formation (Xu et al., 2017). In these previous studies involving *LuxS* mutations, there are different opinions regarding the production of AI-2 and the regulation of methyl metabolism. Therefore, there is still controversy regarding whether the bacterial phenotype changes are caused by QS or disrupted methyl metabolism.

DZ2002 is a type III reversible inhibitor that targets S-adenosylhomocysteine hydrolase (Wu et al., 2023). S-adenosylhomocysteine hydrolase is a key enzyme in the AMC, widely present in mammals, plants and microorganisms (Kusakabe et al., 2015). Therefore, it is necessary to clarify the function and regulatory mechanism of S-adenosylhomocysteine hydrolase in bacteria and human cells in order to perform specific intervention on bacteria when S-adenosylhomocysteine hydrolase is used as a target. Current research indicated that DZ2002 exhibits immunomodulatory properties. For instance, it can reduce the production of pro-inflammatory cytokines by macrophages, alleviate allograft rejection reactions, improve renal function in lupus nephritis, and alleviate symptoms of systemic sclerosis and psoriasis (Wu et al., 2005; He et al., 2019; Zhang et al., 2019; Huang et al., 2020; Chen et al., 2021).

However, there have been no reports on the impact of DZ2002 on bacteria. Considering the importance of SahH in the AMC of *P. aeruginosa*, previous studies may have focused too much on methyl metabolism and AI-2, but neglected the individual impact of SahH on microbial biofilm formation. Therefore, we conducted a series of experiments using DZ2002 to investigate its effects on the formation of mature biofilms by *P. aeruginosa*. The research results indicated that DZ2002 might has a high affinity with SahH in PAO1, and can inhibit the formation of mature biofilms by regulating the levels of alginate, eDNA and swarming motility.

## Materials and methods

### Strains and culture conditions

PAO1 was stored in a ceramic bead culture preservation tube (pythonbio PT001-1) at -70°C in the laboratory. Typically, a single colony was picked from a freshly streaked plate and incubated overnight in Luria-Bertani (LB) broth with shaking (37°C, 200 rpm). The overnight culture was then diluted in LB medium to a concentration of 0.5 MCF (1.5×10^8^ CFU/ml) for subsequent experiments. DZ2002 was purchased from MedChemExpress (HY-18620, China, Shang hai) and prepared as a 100 mM stock solution in DMSO and stored at -80°C for later use. The stock solution was diluted in LB to obtain final working concentrations of 0.01, 0.1, 1, 10, and 100 μM. Bovine pancreatic DNase I (Roche, Switzerland) and RNase (Roche, Switzerland) powders were suspended in deionized water (final concentration is 100 mg/ml) and prepared fresh before use.

### Molecular docking assay

In order to evaluate the binding ability of DZ2002 to PAO1’s SahH, we first downloaded the amino acid sequence of the protein from NCBI, with the serial numbers NP_0006778.1 (Homo sapiens) and AAG03821.1(PAO1), respectively. Then, we use SwissModel for homology modeling of amino acid sequence, and remove protein crystal water, original ligand by Pymol2.3.0. Next, we imported the obtained protein structure into AutoDocktools (v1.5.6) and predicted the active site with POCASA 1.1. The 3D structure of DZ2002 (CAS: 33231-14-0) was downloaded from PubChem and optimized in ChemBio3D Ultra 14.0 before being imported into AutodockTools-1.5.6 for hydrogenation and other processing. Finally, we used AutoDock Vina1.1.2 for docking to evaluate the binding ability of the SahH active site to DZ2002.

### Growth curve determination

The method described in reference (Tan et al., 2019) was slightly modified for this experiment. In brief, the overnight culture of PAO1 was adjusted to a concentration of 0.5 MCF. Subsequently, 1 volume of the overnight PAO1 culture was mixed with 9 volumes of different concentrations of DZ2002 (ranging from 0.01 to 100 μM) and incubated with shaking at 37°C and 200 rpm for 24 hours. Every two hours, 200 μl of the suspension was taken, and the OD570 value was quantified using a spectrophotometer (Multiskan Go microplate reader, Thermo Fisher Scientific, USA). The obtained data was used to plot a time-growth curve to evaluate the inhibitory effects of different concentrations of DZ2002 on the growth of planktonic PAO1.

### Crystal violet staining for biofilm quantification

Simply, minor revisions are made based on these literatures (Sarkar et al., 2014; Gnanadhas et al., 2015), the steps for assessing biofilm biomass using crystal violet staining are as follows: 50 μl of PAO1 and 450 μl of DZ2002 (ranging from 0.01 to 100 μM) were added to a 24-well plate (NEST, China, Guangdong). The plate was then incubated at 37°C for 48 hours to allow the formation of mature biofilms. Subsequently, the planktonic bacteria were gently removed by washing with phosphate-buffered saline (PBS) and left to air dry. At room temperature, 500 μl of 0.1% crystal violet dye was added and incubated for 5 minutes. The unbound dye was washed off with PBS, and the dye absorbed by the biofilms was dissolved with 500 μl of 95% ethanol. The OD570 value was quantified using Multiskan Go microplate reader.

### RT-qPCR assay for detecting expression of gene

The mature biofilms were established in a 12-well plate containing sterilized slides. In brief, DZ2002(10 μM) was added or not added to PAO1, incubated for 48 hours, and then the biofilms were eluted to 1ml PBS using an ultrasonic cleaner (Tomy UD-201, Tokyo, Japan). The eluent was centrifuged and the white precipitation in the lower layer was treated with RNAiso Plus (Takara, Japan) to obtain total RNA. Then, the process of reverse transcription of total RNA into cDNA was performed in A300 Fast Thermal Cycler (LongGene, China) instrument with PrimeScriptTM RT (RR047A, Takara, Japan). RT-qPCR was performed using SYBR® Premix (RR820A, TaKaRa, Japan) on the LightCycler 480 (Roche, Switzerland). Using *rpsl* as the housekeeping genes, the circulating threshold (Ct) of the target gene was calculated using the 2^-△△^ method relative to the Ct of the control group, and it was normalized to the average value of the internal reference gene *rpsl* Ct value of the corresponding sample. Finally, the obtained values were used to assess mRNA expression levels.

### The m-hydroxybiphenyl method for detecting the relative content of alginate

In this experiment, we used the m-hydroxybiphenyl method (Filisetti-Cozzi and Carpita, 1991) to assess the relative content of alginate. Briefly, in a 12-well plate (NEST, China, Guangdong) containing sterilized glass slides, 900 μl of DZ2002 (10 μM) was added to 100 μl of PAO1 suspension for intervention. After 48 hours of static cultivation, mature biofilms were formed. The glass slides were gently washed with PBS to remove planktonic bacteria while retaining the biofilms. Then, the glass slides were placed in a test tube and 3 ml of PBS and 0.6 ml of a mixture of sulfuric acid and sodium tetraborate were added. The test tube was placed in boiling water and boiled for 5 minutes. After cooling, 10 μl of 1% m-hydroxybiphenyl solution was added for color reaction, and absorbance was determined at 520 nm using spectrophotometer.

### Plate motion experiments for assessing swarming motility

In this experiment, we used LB agar plates containing 0.6% agar to assess bacterial swarming motility (Ha et al., 2014). Briefly, 2.5 μl of PAO1 bacterial suspension was carefully inoculated onto the surface of the swarming agar plates. The experiment was divided into two groups (control group and DZ2002 group). DZ2002 group with the presence of DZ2002 (10 μM) and the control group without the presence of DZ2002. The plates were placed upside down and incubated in 5% CO2 incubator for 2 days. After incubation, the area of swarming motility on the plates was quantified using ImageJ software.

### Agarose gel electrophoresis visualizes the extracellular DNA

The detection of eDNA in biofilms was modified based on previous reports (Gnanadhas et al., 2015; Jennings et al., 2015; Tahrioui et al., 2019). Mature biofilms were constructed in a 12-well plate containing glass slides. In the presence or absence of DZ2002 (working concentration of 10 μM), DNase I (working concentration of 100 μg/ml) was added or not added to the LB medium containing PAO1 (0.5 MCF). The biofilms were detached into 1 ml of PBS using an ultrasonic cleaner and centrifuged (2000g, 5 min) to obtain the supernatant. The supernatant was subjected to agarose gel electrophoresis (1.5% agarose) to visualize eDNA. In addition, the eDNA was precipitated from the supernatant using NaCl and ethanol, and finally the eDNA was quantified using a Nanodrop. To confirm that the nucleic acids in the supernatant was eDNA, DNase I or RNase (working concentration of 100 μg/ml) was added to the control group supernatant, followed by incubation at 37°C for 15 minutes before electrophoresis.

### Confocal laser scanning microscopy for observing biofilms and eDNA

As described earlier (Tahrioui et al., 2019), four groups of glass slide biofilms were established in a 12-well plate: DNase I group, DZ2002 group, DNase I and DZ2002 group, and control group. The addition was made with 100 μl of PAO1, 900 μl of the DZ2002, and 1 μl of DNase I. The L7012 LIVE/DEAD™ BacLight™ bacterial viability staining kit (Thermo Fisher Scientific, USA) was used to label the cells. This kit contains two probes: SYTO™ 9 and propidium iodide (PI). The SYTO™ 9 probe (5 μM) was used to detect biofilms, as it can penetrate both live and dead bacteria, while the propidium iodide (PI, 10 μM) probe, which cannot penetrate live bacteria, was used to detect eDNA (Tang et al., 2013; Jennings et al., 2015). The staining was performed in the dark for 15 minutes, and before staining, the biofilms were washed with physiological saline to remove planktonic bacteria. After staining, unbound dye was washed away, and the samples were mounted with 70% glycerol. Fluorescence was observed under a FV3000 CLSM (Olympus, Japan). SYTO™ 9 was excited with 488 nm light, and emission was observed in the range of 500 to 600 nm. PI was excited with 561 nm light, and emission was observed in the range of 570 to 670 nm. For the three-dimensional imaging of biofilm structures, each stack had a thickness of 0.2 μm. The COMSTAT2.1 software (Heydorn et al., 2000) was used for quantitative analysis of the stacks to determine biomass (μm^3^/μm^2^) and average thickness of biomass (μm).

### Statistical analysis

Data analysis was performed using GraphPad Prism 6.01 software. All data were obtained from three independent replicate experiments and are presented as mean ± standard error (SEM). Differences between groups were assessed using two-tailed t-tests or one-way ANOVA. *P*<0.05 was considered statistically significant.

## Results

### Molecular docking assay reveals strong binding affinity between DZ2002 and SahH of PAO1

**Figure 2:**
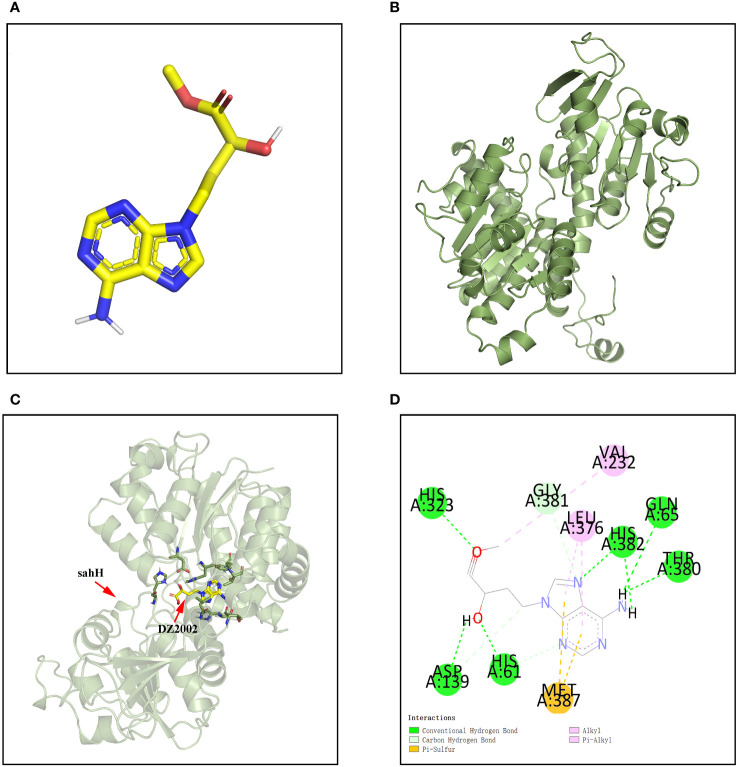
Interaction between DZ2002 and SahH in PAO1. **(A)** The three-dimensional structure of DZ2002. **(B)** The three-dimensional structure of SahH. **(C)** Specific interactions between DZ2002 and SahH in the 3D structural diagram. **(D)** DZ2002 interacts with SahH mainly through hydrogen bonding. The binding energy between the small molecule and PAO1 is -8.7 kcal/mol, indicating a strong binding interaction. The small molecule interacts with SahH primarily through hydrogen bonding and hydrophobic interactions.

The molecular docking was applied to assay the binding affinity between DZ2002 and SahH. The three-dimensional structures of DZ2002 and SahH are shown in **Figures 2A, B**, respectively. The docking result revealed that the DZ2002 is enveloped within the SahH cavity, in close proximity to the enzyme’s active site (**Figure 2C**). In the homotetramer of SahH, each monomer binding one molecule of NAD+, and the central region rich in glycine residues is the binding site for NAD (Sganga et al., 1992; Kloor and Osswald, 2004). The 2D diagram of interactions (**Figure 2D**) revealed that DZ2002 interacts primarily with SahH through hydrogen bonding, predominantly at the NAD binding site. Furthermore, the binding energy analysis indicates a high affinity between DZ2002 and SahH, with a binding energy of -8.7 kcal/mol, compared to -8.6 kcal/mol with human SAHH.

### DZ2002 cannot inhibit the growth of PAO1 within 24h

**Figure 3:**
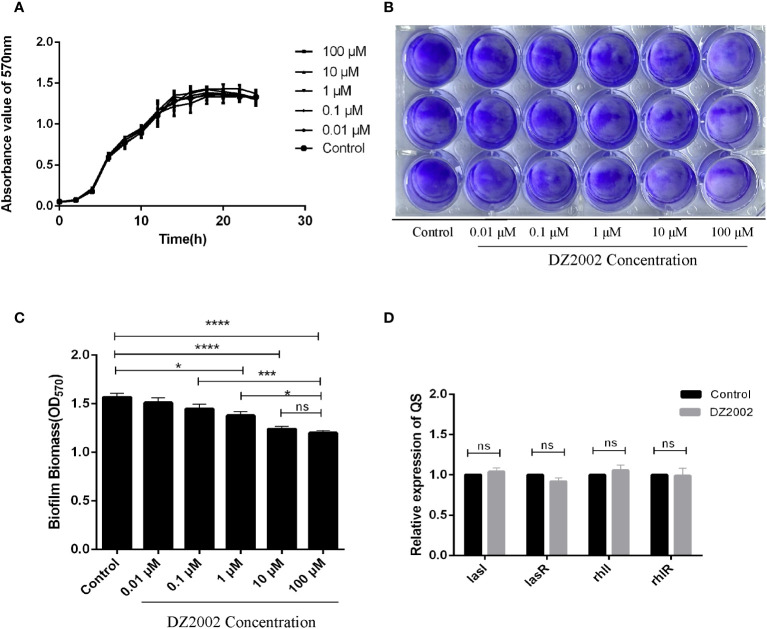
Effect of DZ2002 on the growth characteristics of PAO1. **(A)** Effect of DZ2002 on growth of PAO1 in plankton. The X-axis represents the time line, the Y-axis represents the change of OD values over time in different treatment groups over 24 hours, and the error line represents the standard error of the mean (n=3). **(B)** Effect of DZ2002 on the formation of PAO1 biofilm. Crystal violet staining was used to characterize the biofilm for 48 hours after DZ2002 intervention. **(C)** 95% ethanol was used to eluate the crystal violet dye solution adsorbed on the film, and quantified by enzymoleter. The error line represents the standard error of the mean. One-way analysis of variance was used to examine differences between groups (n=9). ns: *P* > 0.05 (there was no significant difference), *: *P* < 0.05, ***: *P* < 0.001, ****: *P* < 0.0001). **(D)** The role of QS gene *lasI*, *lasR*, *rhII* and *rhIR* mRNA in inhibition of PAO1 biofilm formation by DZ2002. The expression level of QS gene in PAO1 biofilm in the presence or absence of DZ2002 was detected by RT-qPCR. *rpsl* was used as the steward gene, and the relative mRNA expression of target gene was calculated by 2^-△△^ method. The error line represents the standard error of the mean. The two-tailed T-test was used to detect differences between groups (n=3), ns:*P*>0.05 (no significant difference).

From the growth curves (**Figure 3A**), compared to the control group, treatment with different concentrations of DZ2002 could not inhibit the growth of PAO1 within 24 hours. These data was obtained from three independent repeated experiments.

### DZ2002 inhibits the formation of mature biofilms in PAO1

The depth of color represents the content of biofilms. Compared to the control group, the biofilms in the DZ2002 treatment groups were thinner, and the amount of biofilms gradually decreased with the increasing concentrations of DZ2002 (**Figure 3B**). Compared to the control group, biofilms in the 1 μM, 10 μM, and 100 μM DZ2002 groups decreased by 12.05%, 20.89%, and 23.45% respectively (**Figure 3C**). However, there was no significant difference between the content of biofilms of 10 μM and 100 μM DZ2002 groups. Therefore, we selected the 10 μM DZ2002 concentration for further experiments.

### DZ2002 has no effect on the expression of *lasI, lasR, rhII* and *rhIR*

QS is an important factor in regulating biofilm formation in *P. aeruginosa*. To clarify whether the impact of DZ2002 on PAO1 biofilms is associated with QS, we assessed the expression of QS genes (*lasI, lasR, rhII, rhIR*) in the presence or absence of DZ2002. Compared to the control group, there were no significant change in the expression of genes after treatment with 10 μM DZ2002 (**Figure 3D**).

### DZ2002 inhibits alginate synthesis by downregulating the expression of alginate genes

**Figure 4:**
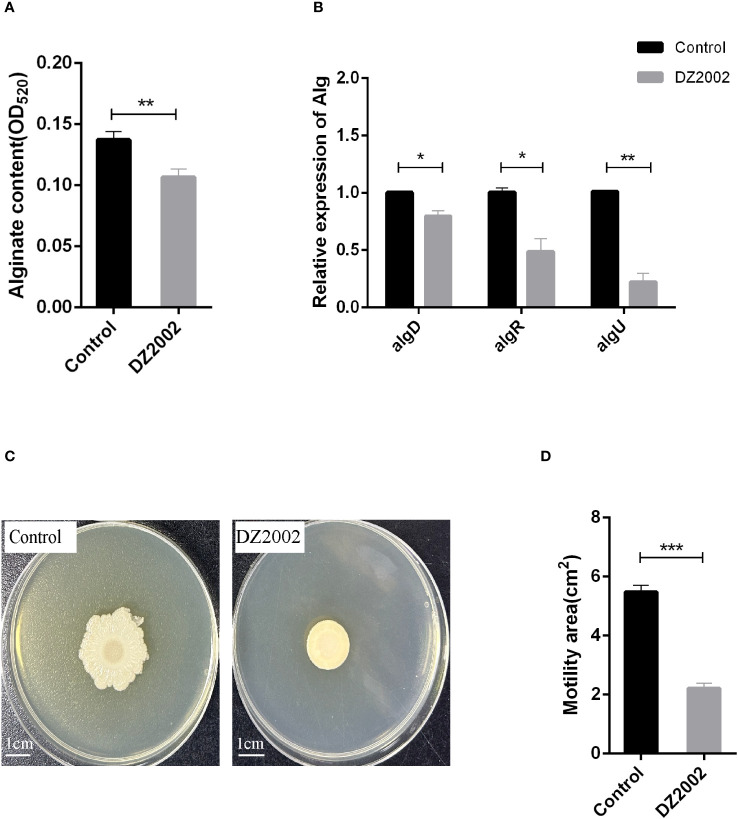
Effect of DZ2002 on alginate in PAO1 biofilm and on the swarming motility of PAO1. **(A)** Effect of DZ2002 on alginate content in PAO1 biofilm. The relative content of alginate in biofilm was measured by m-hydroxy-biphenyl assay after 48 hours culture with or without DZ2002. Error bars represent the standard errors of the mean (SEM). A two-tailed T-test was used to test the difference between groups (n=9), **: *P*<0.01. **(B)** The role of *algD*, *algR*, and *algU* in the inhibition of PAO1 biofilm formation by DZ2002. RT-qPCR was used to measure the expression levels of alginate-related genes in biofilm with or without DZ2002. Using *rpsl* as the housekeeping gene, the relative expression of target gene mRNA was calculated using the 2^-△△^ method. Error bars represent the standard errors of the mean. A two-tailed T-test was used to test the difference between groups (n=3), *: *P*<0.05; **: *P*<0.01. **(C)** The impact of DZ2002 on PAO1 swarming motility. The swarming motility of PAO1 was evaluated on a 0.6% agarose plate with or without DZ2002, with the ruler representing 1 cm. **(D)** The area of swarming motility on the plate was quantified using ImageJ, with the error bars representing the standard errors of the mean (SEM). A two-tailed T-test was used to test the difference between groups (n=6), ***: *P*<0.001.

The results showed that compared to the control group, exposure to 10 μM DZ2002 led to a decrease in alginate content by 22.28% (**Figure 4A**). Similarly, the mRNA expression levels of *algD*, *algR*, and *algU* were significantly downregulated (**Figure 4B**), with reductions of 20.89%, 51.54%, and 77.89%, respectively. These results indicate that DZ2002 inhibits the synthesis of alginate by downregulating the expression of alginate-related genes (*algD, algR, algU*), ultimately suppressing biofilm formation. That is, DZ2002 may reduce alginate content by directly inhibiting alginate synthesis, rather than by indirectly reducing biofilm formation.

### DZ2002 affects swarming motility of PAO1

We attempted to evaluate the effect of DZ2002 on the swarming motility of PAO1 using the agar plate method (**Figure 4C**). Compared to the control group, exposure to 10 μM DZ2002 resulted in a significant reduction in the swarming area of PAO1, with a decrease of 59.54% (**Figure 4D**).

### DZ2002 inhibits the formation of eDNA in PAO1

**Figure 5:**
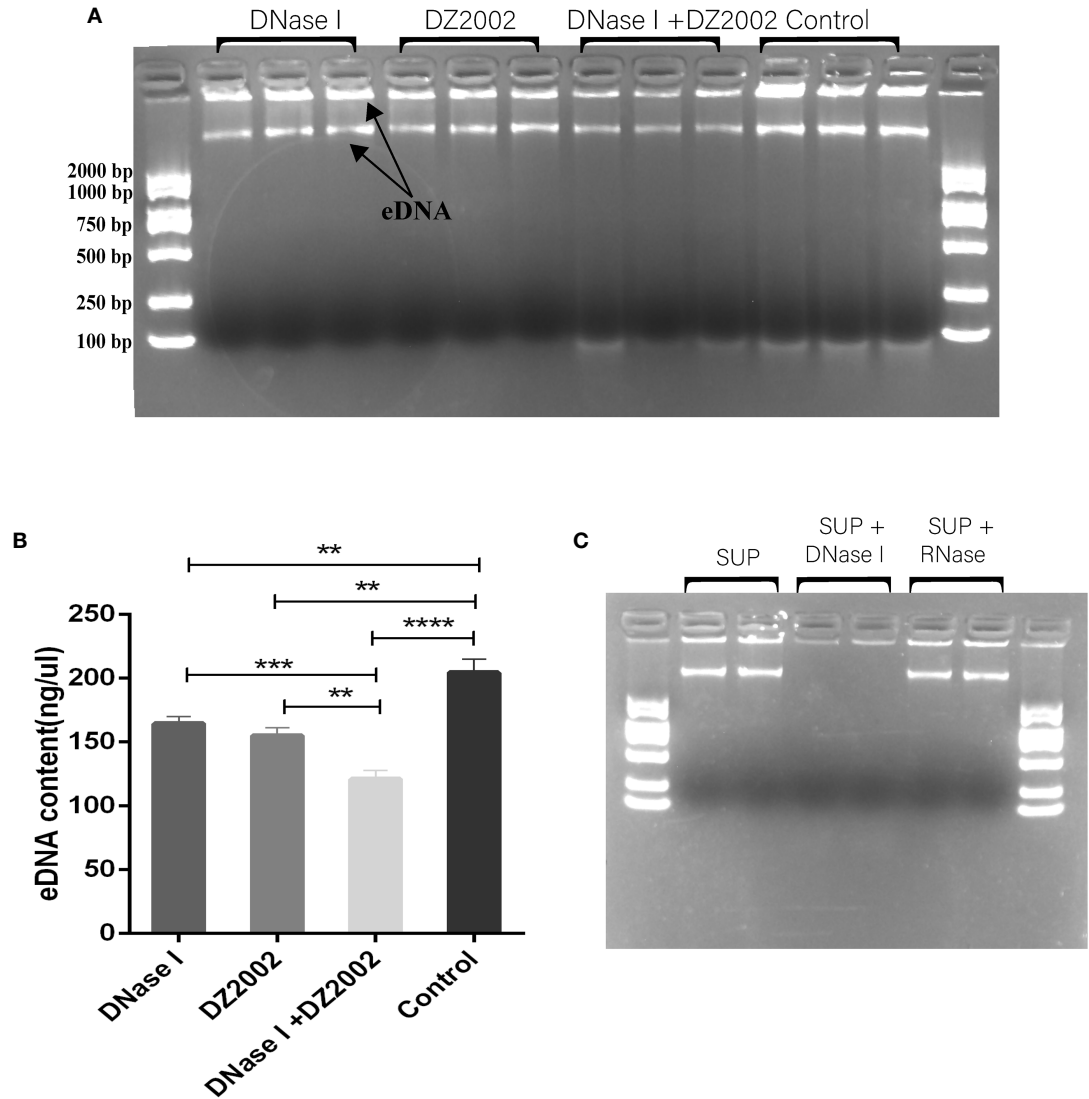
The effect of DZ2002 on extracellular DNA in PAO1 biofilms. **(A)** Visualization of eDNA using 1.5% agarose gel electrophoresis. The arrow represents two bands of eDNA. Electrophoresis for 15 minutes at 170V. **(B)** Quantitative analysis of the eDNA using Nanodrop. Error bars represent the standard error of the mean (SEM). Two-tailed t-test was used to assess intergroup differences (n=6), **: *P*<0.01; ***: *P*<0.001; ***: *P*<0.001; ****: *P*<0.0001. **(C)** Supernatant (SUP) from control biofilms treated with 100 μg/ml DNase I or RNase at 37°C for 15 minutes, then loaded onto a 1.5% agarose gel for visualization.

Previous studies have shown that eDNA acts as a scaffold in biofilms, contributing to their structural stability along with other EPS (Seviour et al., 2021). In this study, we used agarose gel electrophoresis to visualize the eDNA of biofilms. The results revealed that it may be two distinct sizes of eDNA bands on the gel (**Figure 5A**). Using Nanodrop to quantify the quality of eDNA, we found that compared to the control group, the content of eDNA decreased by 19.54% in the DNase I group, 24.08% in the DZ2002 group, and the most significant reduction of 40.72% was observed in the DNase I and DZ2002 combination group (**Figure 5B**). Additionally, we added DNase I or RNase (100 μg/ml) to the control group supernatant and performed electrophoresis after incubation at 37°C for 15 minutes. The results showed a decrease in band intensity in the DNase I group (**Figure 5C**), while no significant change was observed in the RNase group, indicated that the nucleic acid in the supernatant we obtained was DNA and not RNA.

### Visual detection of biofilms and *in situ* eDNA by CLSM

**Figure 6:**
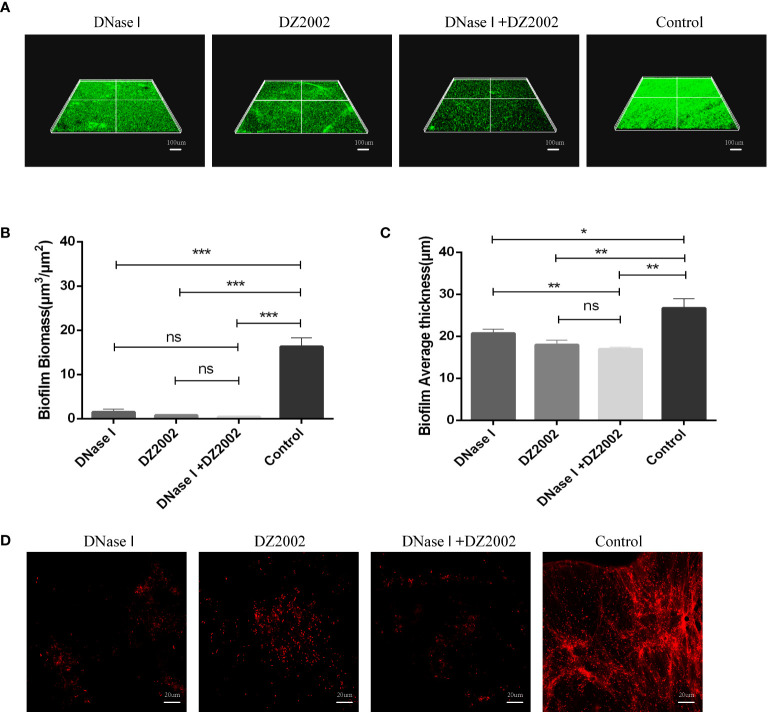
CLSM observes the 48 hours biofilm of PAO1. **(A)** CLSM observation of biofilm biomass. Biofilm was stained with SYTO™ 9, and the fluorescence intensity was observed under a 10X microscope. Living bacteria, dead bacteria and eDNA were stained green. The ruler represents 100 μm. **(B)** Quantification of biofilm biomass was performed using COMSTAT2.1. **(C)** Quantification of the average thickness of biofilms was performed using COMSTAT2.1. Error bars represent the standard errors of the mean (SEM). Two-tailed T test was used to test the differences between groups (n=6), ns: P>0.05 (no significant difference); *: *P*<0.05; **: *P*<0.01; ***: *P*<0.001. **(D)** CLSM observation of *in situ* eDNA in biofilm. After staining biofilm with PI, the fluorescence intensity was observed under a 60X microscope. Dead bacteria and eDNA were stained red. The ruler represents 20 μm.

To further confirm the role of eDNA in inhibiting PAO1 biofilm formation by DZ2002, we employed CLSM to visualize biofilms and *in situ* eDNA. When biofilms were stained with SYTO™ 9, live bacteria, dead bacteria, and eDNA were all stained green. The biofilms of three-dimensional images obtained by CLSM showed that the control group had thicker biofilms and more dense cell clusters compared to the other treatment groups (**Figure 6A**). Quantitative analysis of CLSM results was performed using COMSTAT2.1 software. The content of biofilms in the DNase I group, DZ2002 group, and DNase I -DZ2002 group decreased significantly compared to the control group (**Figure 6B**). Similarly, the average thickness of biofilms showed similar results. Compared to the control group, the average thickness decreased by 22.5%, 32.6%, and 36.4% in the DNase I group, DZ2002 group, and DNase I -DZ2002 group, respectively (**Figure 6C**). Additionally, eDNA in the biofilms was characterized by staining with propidium iodide (PI) dye and visualized using CLSM. Dead bacteria and eDNA were stained red. As expected, the fibrous red fluorescence signal (eDNA) in the biofilms was weakened in the DNase I group, DZ2002 group, and DNase I and DZ2002 group, except for the control group (**Figure 6D**).

## Discussion

The formation of biofilms can effectively assist *P. aeruginosa* colonization, enhance bacterial resistance to antimicrobial agents, as well as the host immune system (Murray et al., 2007). The aforementioned statement represents one of the mechanisms in which bacteria are able to endure in challenging surroundings. Traditional single antibiotic therapy has limitations in treating biofilm infections, such as Tobramycin (Žiemytė et al., 2021). Therefore, more and more studies are exploring novel biofilm inhibitors to treat *P. aeruginosa* biofilm infections. As far as we know, our study is the first to investigate the impact of DZ2002 on the biofilm formation of *P. aeruginosa*.

Molecular docking is a powerful tool that can be used to evaluate the binding affinity between small molecules and proteins, reflecting changes in enzyme activity (Zuo et al., 2023). To test the feasibility of our hypothesis, we conducted molecular docking experiments to assess the interaction between DZ2002 and the predicted active site of S-adenosylhomocysteine hydrolase in both PAO1 and human-derived. We found that DZ2002 exhibited a strong binding affinity to the predicted active site of S-adenosylhomocysteine hydrolase, with binding energies of -8.7 kcal/mol and -8.6 kcal/mol, respectively. This suggested that DZ2002 is likely to bind to PAO1’s SahH and affect its enzymatic activity. Therefore, we evaluated the effects of DZ2002 on the biological characteristics of *P. aeruginosa* in the subsequent experiments.

The formation of biofilms by *P. aeruginosa* involves four distinct stages (Kostakioti et al., 2013), including the initial adhesion (Olivares et al., 2020), irreversible adhesion (Thi et al., 2020), maturation (Johnson et al., 2022; Sauer et al., 2022), and dispersal (Kim and Lee, 2016). Olivares, E et al. (Olivares et al., 2017) discovered that sub-minimum inhibitory concentration (sub-MIC) of tobramycin inhibits *P. aeruginosa* biofilm formation by affecting its early adhesion, which is inconsistent with the results recently described by Tahrioui, A et al. (Tahrioui et al., 2019). Additionally, Žiemytė, M et al. (Žiemytė et al., 2021) found that piperacillin-tazobactam does not exhibit anti-biofilm activity against bacteria at low concentrations. Therefore, the effect of conventional antibiotics on biofilm formation is limited and there are many uncertainties. Compared with the above-mentioned antibiotics, our results indicated that DZ2002 can not affect the growth of planktonic PAO1. However, increasing the concentration of DZ2002 gradually reduces the formation of mature biofilms, as observed through crystal violet staining. To our knowledge, this is the first study to observe the inhibitory effect of DZ2002 on PAO1 biofilm formation. This suggested that DZ2002 has the potential to be a candidate drug for treating biofilm infections. Interestingly, there was no significant difference in biofilm changes between 10 μM and 100 μM DZ2002 groups. Since DZ2002 may bind to the active site of SahH, it is speculated that this lack of difference could be attributed to the fact that 10 μM DZ2002 has already reached the maximum inhibition of SahH activity. Therefore, further investigation is required to understand the mechanism of enzyme activity inhibition.

The mechanisms regulating biofilm formation are extremely complex, and quorum sensing (QS) is crucial (Su and Ding, 2023). We speculated that DZ2002 may exert its anti-biofilm effect by interfering with QS of bacteria. Therefore, we investigated the effect of DZ2002 on PAO1 QS related gene (*lasI, lasR, rhII, rhIR*). Interestingly, our results did not support the hypothesis. This suggested that DZ2002 may exhibit anti-biofilm activity through other mechanisms. In addition, based on the results of molecular docking, QS gene, and crystal violet staining, we speculated that SahH may play a significant role in mediating the formation of PAO1 biofilm by regulating methylation metabolism. However, our findings suggested that this process is independent of QS, as observed elsewhere (Redanz et al., 2012; Hu et al., 2018).

Alginate is a kind of extracellular polysaccharide in biofilm EPS, and its synthesis is regulated by alginate-related genes *algD*, *algR*, *algU* (Okkotsu et al., 2013). Zhao, Z et al. (Zhao et al., 2022) found that when azithromycin combined with berberine interfered with PAO1, the expression of alginate regulatory genes (*algD*, *algR* and *algU*) was reduced in line with alginate content. Consistent with the above study, our study found that the expression levels of *algD*, *algR* and *algU* in 48 hours PAO1 biofilm decreased parallel to alginate content when exposed to DZ2002. In addition, Nivens, D.E et al. (Nivens et al., 2001) discovered that the presence of alginate helps bacteria resist phagocytosis by macrophages under the biofilm. However, the addition of exogenous alginate does not achieve this effect (Rowe et al., 2023). Therefore, we hypothesize that a potential role of DZ2002 is to enhance phagocytosis of biofilm by macrophages, possibly by inhibiting the synthesis of alginate in *P. aeruginosa*. The two-component system FimS-algR can help bacteria sense changes in the external environment and adapt to the environment (Rodrigue et al., 2000). In this complex system, *algR* activation needs to be activated by *algU* dependent pathway, and finally activate *algD* operon to complete the process of alginate overexpression in mucoid *P. aeruginosa* (Deretic et al., 1989; Ma et al., 1997; Ma et al., 1998). For non-mucoid *P. aeruginosa*, during infections, the immune system induces MucA protein hydrolysis and release of *algU* (Damron and Goldberg, 2012), which activates *algD* operon and then promotes *algR* (Darzins and Chakrabarty, 1984) expression and alginate synthesis. Therefore, the expression regulation of *algD*, *algR*, and *algU* is crucial for the transition between mucous and non-mucoid phenotypes. In cystic fibrosis patients, mucous phenotypes are often present due to excessive alginate production (Govan and Deretic, 1996). In our study, DZ2002 showed good inhibitory effects on the expression of *algD*, *algR*, *algU*, and alginate synthesis, indicating that DZ2002 will be an ideal drug for the prevention and treatment of mucous and non-mucoid *P. aeruginosa* infections. In addition, these results further confirmed the inhibitory effect of DZ2002 on the formation of mature biofilms of PAO1.

In *P. aeruginosa*, *algR* can not only regulate the production of alginate, but also guide the movement of bacteria and participate in the formation and diffusion of biofilms and the generation of drug resistance (Cai et al., 2020; Coleman et al., 2020). It has been reported that *algR* can affect not only the alginate production of *P. aeruginosa*, but also the ability to swarm (Whitchurch et al., 1996). Similarly, Our study found that DZ2002 not only inhibited the expression of genes such as *algR* and alginate formation, but also significantly limited the swarming motility of bacteria (reduced by 59.54%), in agreement with the results recently discovered by Okkotsu, Y et al. (Okkotsu et al., 2013). Therefore, we speculate that the effect of DZ2002 on PAO1 swarming may be related to the downregulation of *algR*, and thus participate in the inhibition of biofilm.

eDNA can interact with many components in EPS to enhance the stability of biofilm structure. Studies have shown that eDNA can form PsI-eDNA fiber webs with extracellular polysaccharide PsI (Byrd et al., 2009), and in addition, eDNA can interact with Pel (Jennings et al., 2015; Jennings et al., 2021) to protect eDNA from degradation by DNase I. Due to the important position of eDNA in biofilm EPS (Kanampalliwar and Singh, 2020; Peng et al., 2020; Dawson et al., 2021; Cassin et al., 2023), more and more scholars have begun to pay attention to drug development targeting eDNA. Previous studies have reported the effects of natural compounds (Meto et al., 2020), synthetic novel substances (Patel et al., 2020) and amino acids (Gnanadhas et al., 2015) on eDNA in *P. aeruginosa* biofilms. Our AGE results demonstrated that the eDNA present in the biofilm may consists of two sizes of DNA molecules. The larger molecular weight eDNA may be genomic DNA released from cell death, as mentioned in previous studies (Tahrioui et al., 2019). The smaller molecular weight eDNA may be extracellular DNA fragments released through active secretion or passive leakage, as observed elsewhere (Deng et al., 2020). However, further research is necessary to investigate the specific information about these two molecular weights of eDNA in biofilms. DNase I has been widely used to validate the importance of eDNA for biofilm formation. For instance, Gnanadhas DP et al. (Gnanadhas et al., 2015) found that DNase reduced the release of eDNA and inhibited *P. aeruginosa* biofilm formation. In this study, we observed by AGE that both DZ2002 and DNase I reduced eDNA production. This reduction in eDNA content correlated with changes in biofilm biomass and thickness observed through CLSM. The results indicated that the decrease in eDNA content caused by DZ2002 correlated with a reduction in biofilm formation in PAO1, as observed elsewhere (Wongkaewkhiaw et al., 2020). Overall, the findings suggested that DZ2002 can inhibit eDNA production and suppress the formation of mature biofilms in PAO1. However, it should be noted that while eDNA is a major pro-inflammatory factor in *P. aeruginosa* biofilms and plays a crucial role in the development of inflammation (Fuxman Bass et al., 2010), the research findings demonstrated that DZ2002 can inhibit eDNA production and suppress the formation of mature biofilms in PAO1. This further highlights the unexpected benefits of DZ2002 as a biofilm inhibitor.

In addition, previous studies have shown (Liu et al., 2020) that DNase I can degrade eDNA, promote the penetration of antibiotics, and increase bacterial sensitivity to antibiotics. Consistent with previous studies, we found that the combination of DZ2002 and DNase I exhibited a stronger inhibitory effect, possibly due to the digestion of eDNA by DNase I, which facilitated the penetration of DZ2002 and enhanced its inhibitory effect on the biofilms. When using CLSM to observe *in situ* eDNA, we have found that DZ2002 and DNase I can cause the disappearance of eDNA fibers in biofilms, as mentioned in previous studies (Seviour et al., 2021). This finding further demonstrated that DZ2002 can inhibit the production of eDNA and suppress biofilm formation. In summary, all of these findings contribute to a deeper understanding of the mechanism of biofilm formation and provide a theoretical basis for the development of novel eDNA inhibitors.

## Conclusion

In conclusion, we found for the first time that DZ2002 can down-regulate the expression of alginate gene in biofilm, inhibit alginate synthesis and swarming motility, and inhibit the synthesis of eDNA, showing good inhibitory activity on the maturation of PAO1 biofilms *in vitro*, but the generation of these phenotypes may not be related to the expression of *lasI, lasR, rhII, rhIR*. In addition, molecular docking results indicate that DZ2002 may play the above role by binding to PAO1’s SahH. It is still necessary to further explore the specific mechanism of alginate and eDNA synthesis regulated by DZ2002 in order to clarify the mechanism of inhibiting biofilm by DZ2002. Due to the complex and changeable environment *in vivo*, it is necessary to further evaluate the effect of DZ2002 on biofilm *in vivo*.

## Data availability statement

The original contributions presented in the study are included in the article/**Supplementary Material**, further inquiries can be directed to the corresponding author/s.

## Author contributions

JD: Investigation, Methodology, Data curation, Writing – original draft. WL: Investigation, Methodology, Funding acquisition, Project administration, Writing – review & editing. FH: Writing – review & editing. SL: Writing – review & editing.
